# Interactions between sucrose and jasmonate signalling in the response to cold stress

**DOI:** 10.1186/s12870-020-02376-6

**Published:** 2020-04-22

**Authors:** Astrid Wingler, Verónica Tijero, Maren Müller, Benqi Yuan, Sergi Munné-Bosch

**Affiliations:** 1 School of Biological, Earth & Environmental Sciences and Environmental Research Institute, University College Cork, Distillery Fields, North Mall, Cork, Ireland; 2 Department of Evolutionary Biology, Ecology and Environmental Sciences, University of Barcelona, Avinguda Diagonal 643, 08028 Barcelona, Spain; 3 Present address: Key Laboratory of Ecology and Environmental Science in Guangdong Higher Education, School of Life Science, South China Normal University, Guangzhou, 510631 China

**Keywords:** Anthocyanin, *Arabidopsis thaliana*, Cold acclimation, Gibberellin, Plant hormones, Jasmonate, Stress response, Sugar signalling

## Abstract

**Background:**

Jasmonates play an important role in plant stress and defence responses and are also involved in the regulation of anthocyanin synthesis in response to sucrose availability. Here we explore the signalling interactions between sucrose and jasmonates in response to cold stress in Arabidopsis.

**Results:**

Sucrose and cold treatments increased anthocyanin content additively. Comprehensive profiling of phytohormone contents demonstrated that jasmonates, salicylic acid and abscisic acid contents increased in response to sucrose treatment in plants grown on agar, but remained considerably lower than in plants grown in compost. The gibberellin GA_3_ accumulated in response to sucrose treatment but only at warm temperature. The role of jasmonate signalling was explored using the jasmonate response mutants *jar1–1* and *coi1–16*. While the *jar1–1* mutant lacked jasmonate-isoleucine and jasmonate-leucine, it accumulated 12-*oxo*-phytodienoic acid at low temperature on agar medium. Altered patterns of abscisic acid accumulation and higher sugar contents were found in the *coi1–16* mutant when grown in compost. Both mutants were able to accumulate anthocyanin and to cold acclimate, but the *jar-1-1* mutant showed a larger initial drop in whole-rosette photosystem II efficiency upon transfer to low temperature.

**Conclusions:**

Hormone contents are determined by interactions between temperature and sucrose supply. Some of these effects may be caused indirectly through senescence initiation in response to sucrose availability. During cold stress, the adjustments of hormone contents may compensate for impaired jasmonate signalling, enabling cold acclimation and anthocyanin accumulation in Arabidopsis jasmonate response mutants, e.g. through antagonistic interactions between gibberellin and jasmonate signalling.

## Background

Exposure to low, but above-zero temperatures activates physiological changes that result in cold acclimation in cold-hardy plants such as Arabidopsis. This process involves hormone signalling [1], in addition to changes in primary metabolism that lead to the accumulation of sugars [2, 3]. There is increasing evidence that the synthesis of sucrose is directly related to cold tolerance: Plants over-expressing sucrose phosphate synthase showed increased freezing tolerance after cold acclimation [4]. Normally, cold acclimation requires light, but external supply of sucrose improved cold acclimation even in the dark [5]. Natural genetic variation of Arabidopsis accessions showed that sugar contents, including sucrose, were correlated with freezing tolerance of cold-acclimated plants [6, 7]. In addition, we have recently shown that sucrose accumulation in response to cold treatment is correlated with altitude of origin in the alpine perennial *Arabis alpina*, suggesting that adaptation to low temperature in alpine plants is related to the capacity to accumulate sucrose [8].

Jasmonates, a group of plant hormones that are involved in stress and defence responses [9], accumulate during cold treatment, and treatment with external methyl jasmonate (MeJA) can increase freezing tolerance [10, 11]. In addition, jasmonic acid (JA) content was positively correlated with that of sucrose in *A. alpina* accessions [8] which is in agreement with the observations that JA accumulation in response to stress in Arabidopsis seedlings is sugar dependent [12].

Both, sucrose availability and cold stress, are typically accompanied by anthocyanin accumulation. It has been proposed that anthocyanins protect the photosynthetic apparatus, most likely acting as sunlight filters, although they have also been proposed to act as antioxidants [13, 14]. The contents of two out of six anthocyanins were correlated with cold-acclimated freezing tolerance in a range of Arabidopsis accessions [15]. Work with anthocyanin synthesis mutants has provided direct evidence for the importance of anthocyanin formation in the response to low temperature. The fitness of these mutants is reduced under favourable conditions and also under cold stress, but not under other forms of stress [16].

Sucrose induces anthocyanin accumulation by activating expression of the MYB transcription factor gene *MYB75* (=*PAP1*; *Production of Anthocyanin Pigment 1*) [17, 18]. However, expression of *MYB75* by low temperature was variable in Arabidopsis accessions, and it is more likely that MYB90 (=PAP2) instead of MYB75 is the main cold-responsive regulator of anthocyanin formation [15]. Sucrose-dependent induction of anthocyanin synthesis is enhanced by MeJA and abscisic acid (ABA) but reduced by gibberellins (GAs) [19]. While MeJA on its own does not induce anthocyanin synthesis or *MYB75* and *MYB90* expression, expression of both transcription factors and accumulation of anthocyanins are enhanced by MeJA treatment in the presence of sucrose [19].

Jasmonate signalling has been explored by selecting mutants that are insensitive to MeJA, resulting in the isolation of *jar1* (*jasmonate resistant 1*) mutants [20], which were later shown to be impaired in the formation of the active JA conjugate JA-Ile [21, 22]. In addition, *coi1* (*coronatine insensitive 1*) mutants that are resistant to the phytotoxin coronatine were identified to lack responses to MeJA [23]. COI1 forms part of the SCF^COI1^ ubiquitin-ligase complex [24] responsible for ubiquitin-dependent protein degradation of jasmonate ZIM-domain (JAZ) proteins, which repress jasmonate function [9]. JAZ proteins are repressors of transcription factors, such as MYC2, that stimulate the expression of jasmonate-responsive genes [25, 26]. Jasmonate signalling via the COI1 pathway requires formation of JA-Ile by JAR1 for interaction between SCF^COI1^ and JAZ1, whereas JA itself or MeJA do not promote this interaction [21, 27].

*coi1* mutants do not accumulate anthocyanins in response to MeJA treatment [19, 23, 28, 29]. However, they were described to accumulate anthocyanins under continuous white light or during drought stress [23]. In contrast to *coi1* mutants, the *jar1–1* mutant accumulates anthocyanins in response to jasmonate [30], and expression of anthocyanin synthesis genes is unaffected by the *jar1–1* mutation [19]. Jasmonate response mutants were also used to determine the role of the JA-Ile signalling during stress, showing that the *jar1–1* and *coi1–16* mutant alleles were both impaired in drought-induced ABA accumulation [31] and hypersensitive to ozone [32].

Crosstalk between jasmonate and GA signalling regulates anthocyanin formation through sequestration of JAZ proteins by DELLA proteins which are repressors of GA signalling. GA-induced degradation of DELLA proteins can release JAZ proteins and thereby repress jasmonate-dependent anthocyanin synthesis [33–35]. In turn, a decline in GA could increase anthocyanin formation by sequestering JAZ proteins and thereby increase anthocyanin synthesis downstream of JA-Ile signalling.

Here, we explore interactive effects between above-freezing cold (i.e. chilling) and sucrose treatments on anthocyanin content in wild-type Arabidopsis and in the *jar1–1* and *coi1–16* mutants. Hormonal profiles were analysed by UHPLC/ESI-MS/MS to evaluate possible hormonal interactions. Furthermore, we determined the effect of cold temperature on photosystem II efficiency to determine the ability of the jasmonate signalling mutants to cold acclimate.

## Results

### Effects of cold and sucrose treatments on F_v_/F_m_ and anthocyanin content

**Fig. 1:**
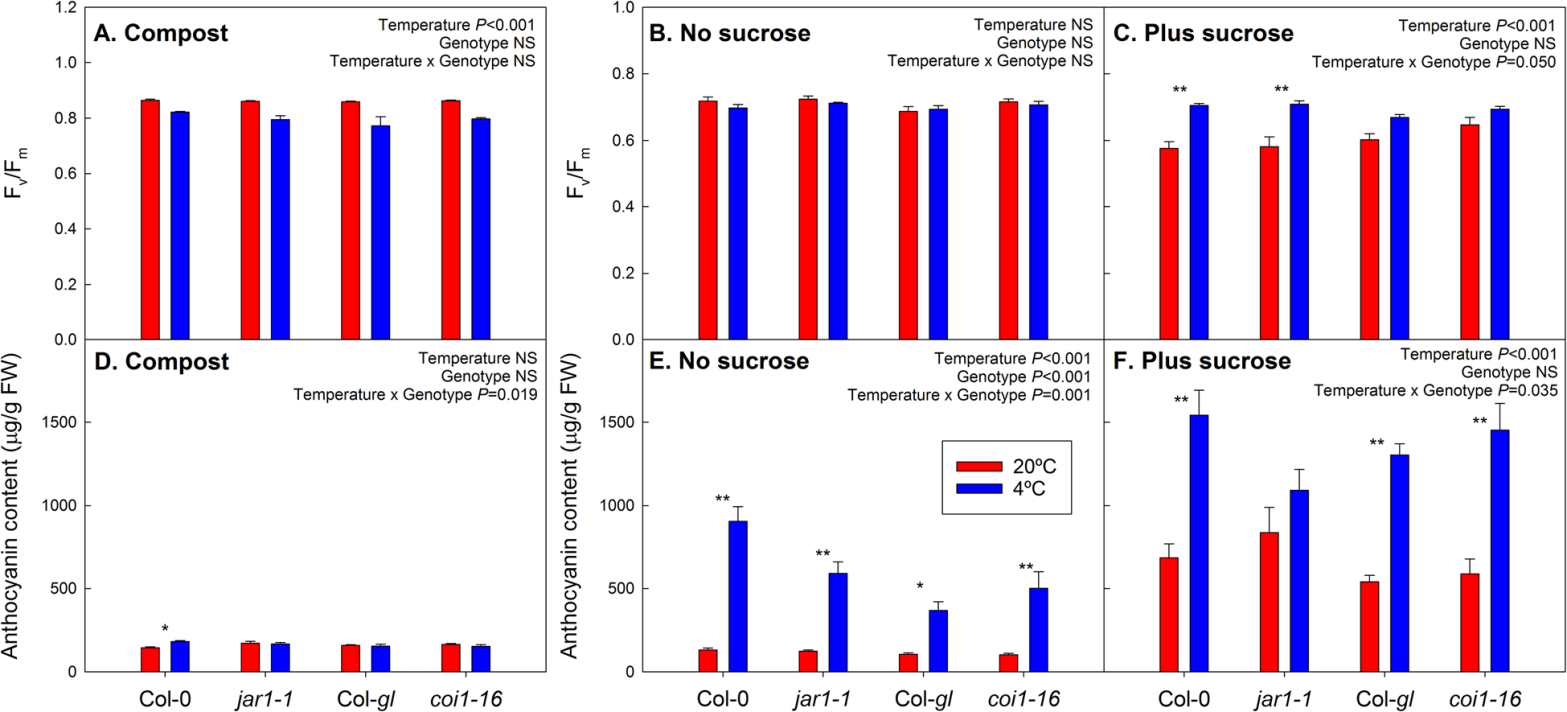
Effect of cold and sucrose treatment on F_v_/F_m_ (**a**-**c**) and anthocyanin content (**d**-**f**) in the *jar1–1* and *coi1–16* mutants and their respective wild types, Col-0 and Col-*gl*. The plants were grown at 20 °C (red bars) or 4 °C (blue bars) in compost (**a** and **d**), or on agar without (**b** and **e**) or with (**c** and **f**) addition of 55.5 mM sucrose. Individual leaves (leaf position 6) were analysed for compost-grown plants and whole shoots for agar plates. Compost-grown plants were harvested after 15 days of temperature treatment (age of plants 54 days) for anthocyanin analysis; agar-grown plants were harvested after 25 days of treatment (age of plants 38 days). F_v_/F_m_ was measured on the day before the harvest. Data are means of 5 plants (compost) or plants from 5 plates +SE. Temperature effects, genotype effects and interactions were analysed by two-way ANOVA. The asterisks indicate statistically significant differences between the 20 °C and 4 °C treatments for each genotype (Tukey’s HSD post-hoc test; * *P* < 0.05; ** *P* < 0.01). To allow comparison of the anthocyanin contents on different growth media, the y-axis scaling is the same for all media

In compost-grown plants, cold treatment led to a slight reduction in F_v_/F_m_ in leaf six of all genotypes, but no significant genotype-specific effects (Fig. 1a). A timecourse of the cold response until the date of harvest is shown in Additional file 1, demonstrating that, while F_v_/F_m_ dropped in response to cold treatment, no senescence-dependent decline in F_v_/F_m_ occurred during the course of the experiment. On agar without sucrose, no significant effect of temperature on F_v_/F_m_ was found (Fig. 1b), probably because the plants had fully acclimated after 25 days of cold treatment, as demonstrated by the recovery of F_v_/F_m_ after the initial drop (Additional file 2). On sucrose-containing medium, F_v_/F_m_ was reduced at warm instead of cold temperature (Fig. 1c). This drop in F_v_/F_m_, which is also evident in the time course (Additional file 2), is consistent with sugar-induced senescence at warm [36], but not cold temperature [37]. No differences in F_v_/F_m_ were detected between the jasmonate response mutants and wild-type plants.

While cold treatment only increased anthocyanin content in Col-0 plants grown in compost (Fig. 1d), there was a strong overall increase in anthocyanin content in response to cold treatment after growth on agar plates, without (Fig. 1e) and with (Fig. 1f) sucrose (*P* < 0.001). While this temperature effect was not statistically significant in *jar1–1* in the presence of sucrose, anthocyanins accumulated in all genotypes (including *jar1–1*) after sucrose treatment compared to agar medium without sucrose (Additional file 3). Strong sucrose effects on anthocyanin content were found at warm and cold temperature (*P* < 0.001; Additional file 4), but without significant interactions between sucrose and temperature treatments that would have indicated synergistic effects. There were no genotype effects on anthocyanin accumulation, other than on agar without sucrose. Normal contents of anthocyanins in the mutants were supported by an independent experiment with compost-grown plants (Additional file 5).

### Effects of cold and sucrose treatments on sugar contents

**Fig. 2:**
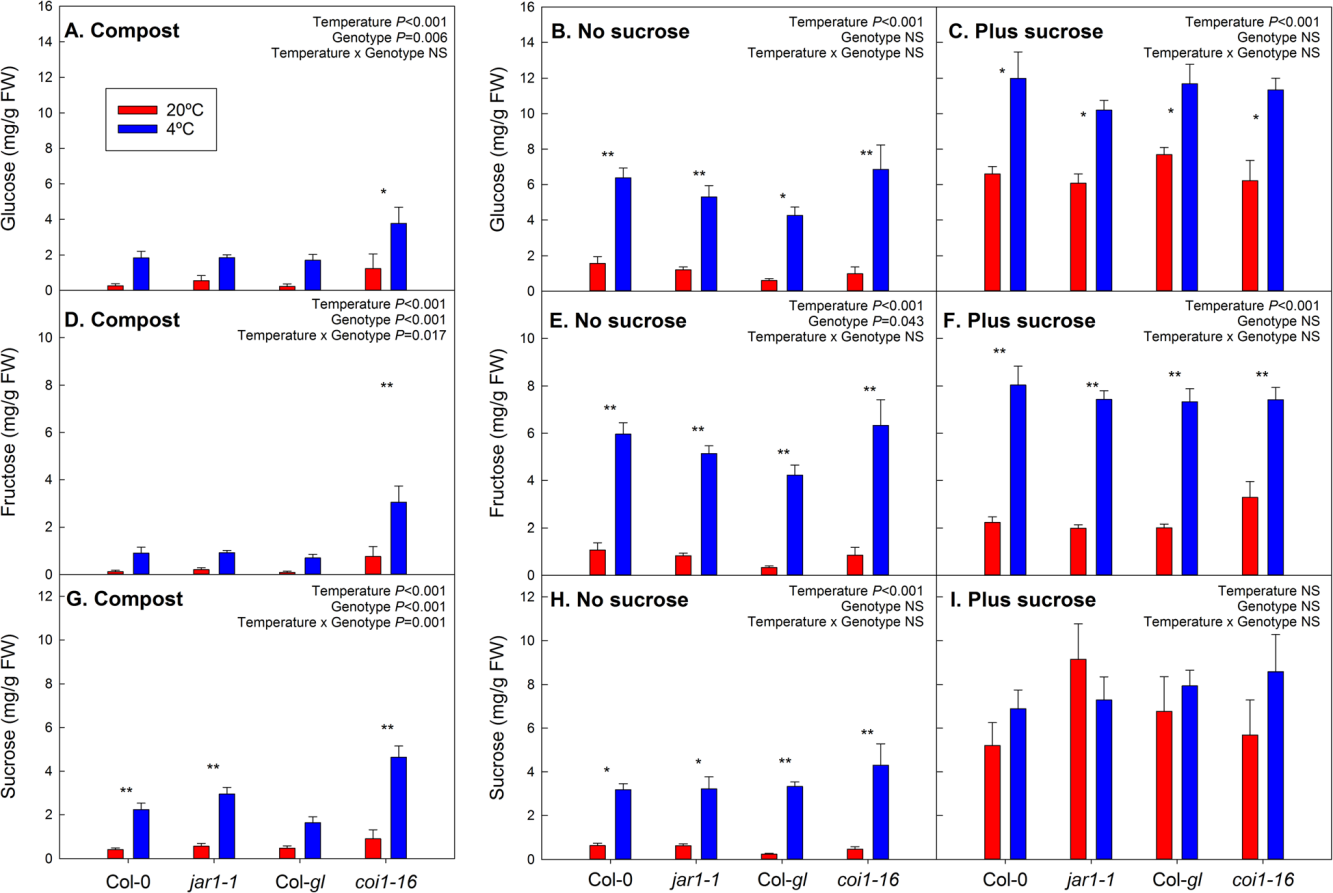
Effect of cold and sucrose treatment on glucose (**a**-**c**), fructose (**d**-**f**) and sucrose (**g**-**i**) contents in the *jar1–1* and *coi1–16* mutants and their respective wild types, Col-0 and Col-*gl*. The plants were grown at 20 °C (red bars) or 4 °C (blue bars) in compost (**a**, **d** and **g**), or on agar without (**b**, **e** and **h**) or with (**c**, **f** and **i**) addition of 55.5 mM sucrose. Individual leaves were analysed for compost-grown plants and whole shoots for agar plates. Compost-grown plants were harvested after 15 days of temperature treatment (age of plants 54 days); agar-grown plants were harvested after 21 days of treatment (age of plants 34 days). Data are means of 5 plants (compost) or plants from 5 plates +SE. Temperature effects, genotype effects and interactions were analysed by two-way ANOVA. The asterisks indicate statistically significant differences between the 20 °C and 4 °C treatments for each genotype (Tukey’s HSD post-hoc test; * *P* < 0.05; ** *P* < 0.01). To allow comparison of the sugar contents on different growth media, the y-axis scaling is the same for all media

As expected, the contents of glucose, fructose and sucrose were increased when sucrose was added to the medium compared to plants on agar without sucrose or plants on compost, especially at warm temperature (Fig. 2), but effects on the contents all three sugars were also highly significant at cold temperature (*P* < 0.001; Additional file 4). In response to cold treatment, glucose and fructose contents accumulated in compost-grown plants and on agar plates with or without sucrose addition (Fig. 2a-f; *P* < 0.001). Sucrose content increased in the cold in the absence of external sucrose supply (Fig. 2g, h; *P* < 0.001), but was already high at warm temperature in the presence of sucrose and did not rise further (Fig. 2i). In compost, the *coi1–16* mutant had significantly higher sugar contents (glucose, fructose and sucrose) than the other lines (*P* < 0.05), and the response to cold temperature was more pronounced than in its Col-*gl* wild-type background (Fig. 2a, d and g). Across treatments and genotypes, all three sugars were strongly correlated with anthocyanin content (*P* < 0.001; Additional file 6).

### Effects of growth conditions on hormone contents

**Fig. 3:**
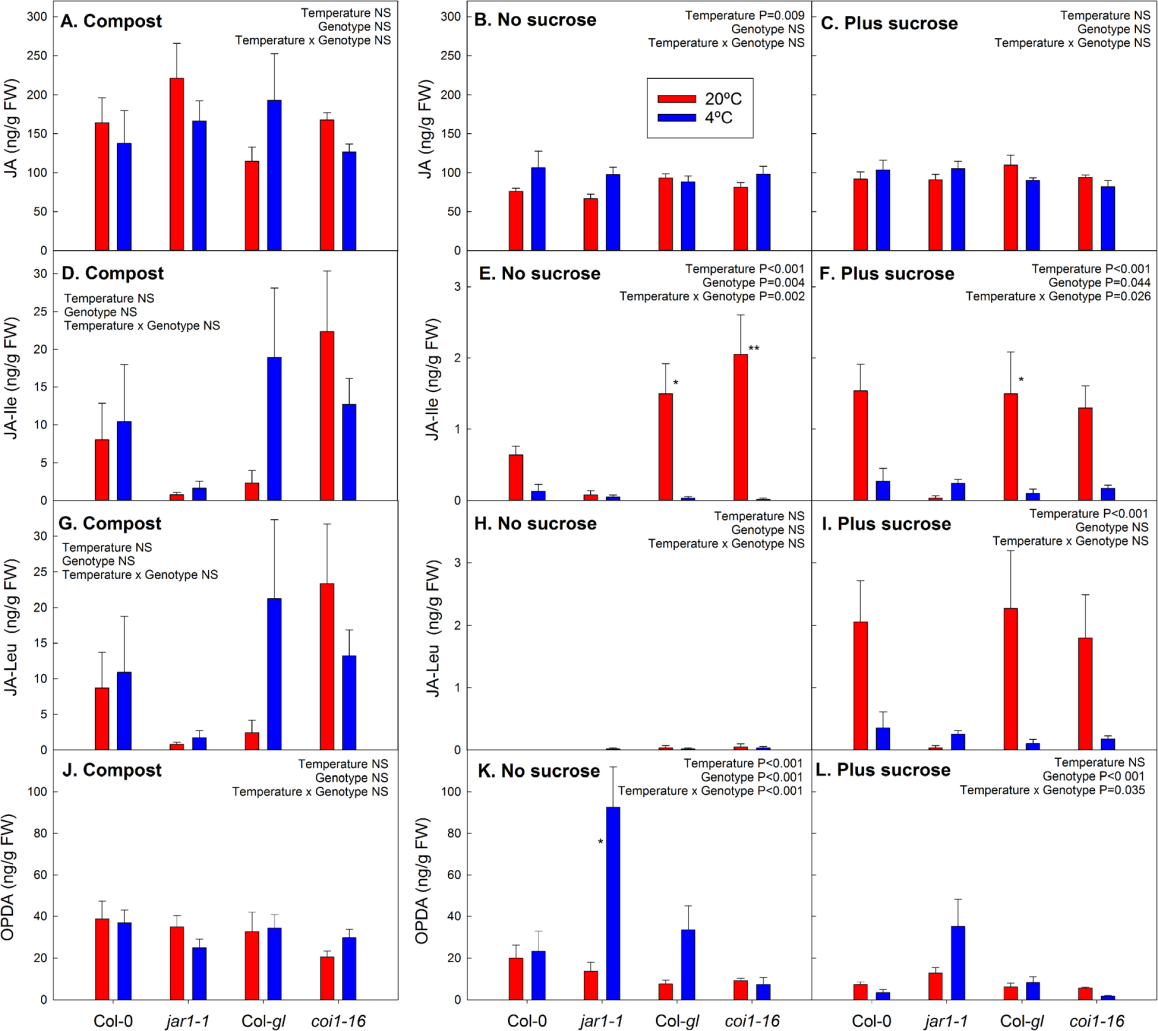
Effect of cold and sucrose treatment on JA (**a**-**c**), JA-Ile (**d**-**f**), JA-Leu (**g**-**i**) and OPDA (**j**-**l**) contents in the *jar1–1* and *coi1–16* mutants and their respective wild types, Col-0 and Col-*gl*. The plants were grown at 20 °C (red bars) or 4 °C (blue bars) in compost (**a**, **d**, **g** and **j**), or on agar without (**b**, **e**, **h** and **k**) or with (**c**, **f**, **i** and **l**) addition of 55.5 mM sucrose. Individual leaves were analysed for compost-grown plants and whole shoots for agar plates. Compost-grown plants were harvested after 15 days of temperature treatment (age of plants 54 days); agar-grown plants were harvested after 25 days of treatment (age of plants 38 days). Data are means of 5 plants (compost) or plants from 5 plates +SE. Temperature effects, genotype effects and interactions were analysed by two-way ANOVA. The asterisks indicate statistically significant differences between the 20 °C and 4 °C treatments for each genotype (Tukey’s HSD post-hoc test; * *P* < 0.05; ** *P* < 0.01). Note that JA-Ile and JA-Leu contents in plants on agar are shown using different y-axis scaling compared to those on compost

Sucrose addition significantly increased the contents of JA (*P* = 0.002) and JA-Leu (*P* < 0.001) at warm temperature and those of JA-Ile (*P* = 0.020) and JA-Leu (*P* = 0.007) at cold temperature (Additional file 4). At warm temperature, the effect of sucrose on the contents of these hormones may be related to early senescence (as indicated by the drop in F_v_/F_m_ values; Fig. 1c and Additional file 2). However, in compost-grown plants, the contents of jasmonates, in particular the conjugates JA-Ile and JA-Leu, were much higher than in plants grown on agar (Fig. 3; note different y-axis scaling for compost- and agar-grown plants). There was also an effect of temperature on JA conjugates: JA-Ile was reduced by cold temperature in the absence of sucrose (*P* < 0.001) (Fig. 3e), and, in the presence of sucrose, both JA-Ile and JA-Leu contents were decreased (*P* < 0.001) (Fig. 3f, i). On compost, temperature effects were less consistent, but in *coi1–16* the effect on JA-Ile and JA-Leu was opposite to its Col-*gl* wild type, although these temperature responses were not statistically significant (Fig. 3d, g).

Our analysis confirmed that the *jar1–1* mutant is deficient in JA-Ile as expected (Fig. 3d-f). Equally, JA-Leu content was lower in *jar1–1* than in the other genotypes after growth in compost (Fig. 3g). This confirms that the *jar1–1* mutant is impaired in the formation of JA-Ile, but also suggests impaired formation of JA-Leu. On agar medium without sucrose, OPDA content in the *jar1–1* mutant was significantly increased in response to cold temperature. OPDA content was higher in *jar-1-1* compared to the other genotypes at cold temperature on agar medium (Fig. 3k, l). However, in compost-grown plants no effects of the mutations or the temperature treatment on OPDA were found (Fig. 3j). No clear trends in JA content (Fig. 3a-c) or in the conjugates JA-Phe and JA-Met (Additional file 7) in response to temperature were detected. JA-Val was always below the detection limit (data not shown).

**Fig. 4:**
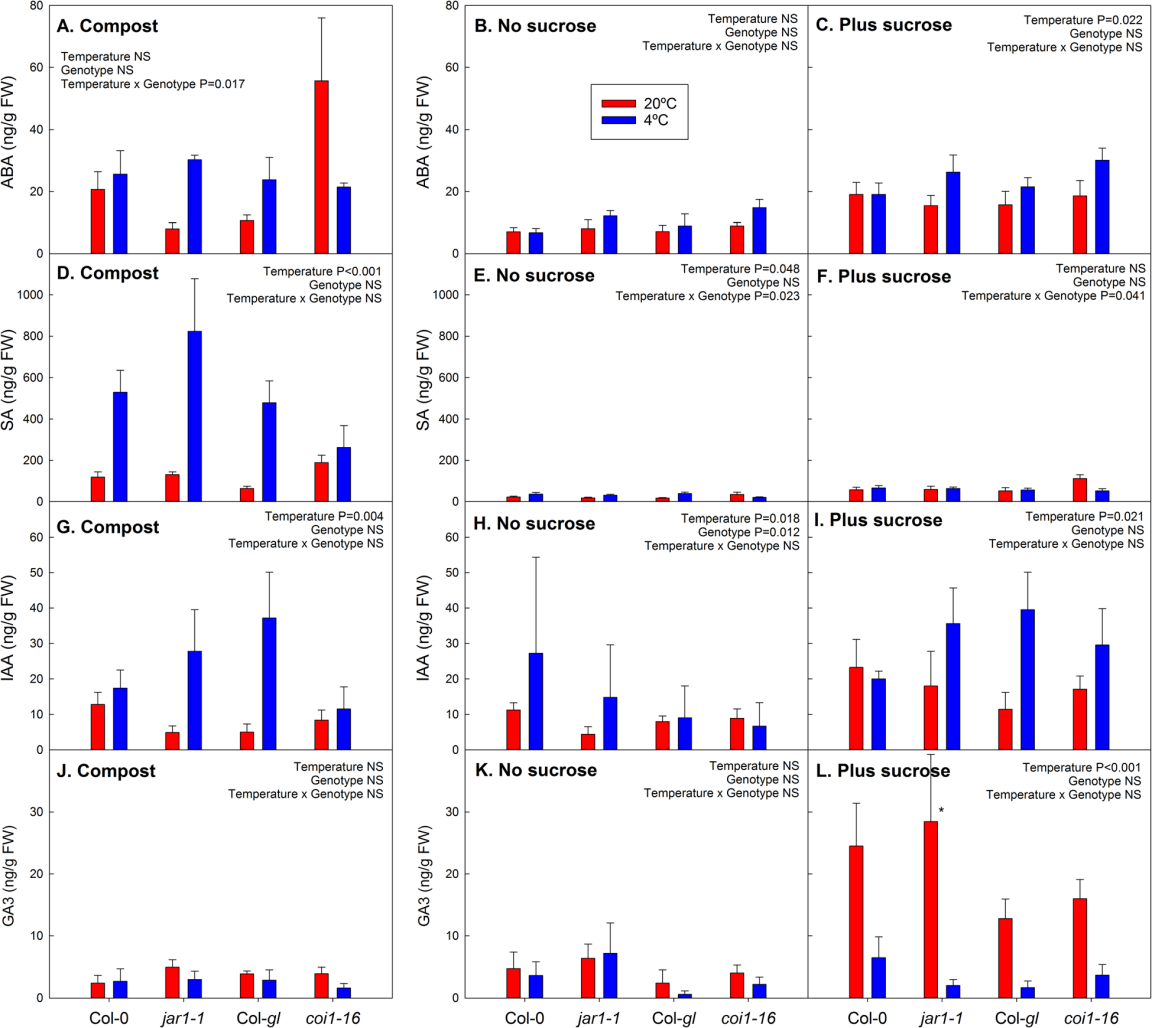
Effect of cold and sucrose treatment on ABA (**a**-**c**), SA (**d**-**f**), IAA (**g**-**i**) and GA_3_ (**j**-**l**) contents in the *jar1–1* and *coi1–16* mutants and their respective wild types, Col-0 and Col-*gl*. The plants were grown at 20 °C (red bars) or 4 °C (blue bars) in compost (**a**, **d**, **g** and **j**), or on agar without (**b**, **e**, **h** and **k**) or with (**c**, **f**, **i** and **l**) addition of 55.5 mM sucrose. Individual leaves were analysed for compost-grown plants and whole shoots for agar plates. Compost-grown plants were harvested after 15 days of temperature treatment (age of plants 54 days); agar-grown plants were harvested after 25 days of treatment (age of plants 38 days). Data are means of 5 plants (compost) or plants from 5 plates +SE. Temperature effects, genotype effects and interactions were analysed by two-way ANOVA. The asterisks indicate statistically significant differences between the 20 °C and 4 °C treatments for each genotype (Tukey’s HSD post-hoc test; * *P* < 0.05; ** *P* < 0.01). To allow comparison of the hormone contents on different growth media, the y-axis scaling is the same for all media

ABA and SA contents were correlated with JA, JA-Leu and JA-Ile, suggesting common regulation of these stress hormones (Additional file 6). Similar to the jasmonates, ABA and SA contents were higher on compost than on agar medium, but also increased by sucrose at warm and cold temperature (Fig. 4a-f; *P* < 0.001; Additional file 4). In addition, SA accumulated at cold temperature on compost (*P* < 0.001). There was a general trend for increased ABA content at low temperature, but, similar to results for JA-Ile and JA-Leu, the *coi1–16* mutant showed an unusual response of ABA to the temperature treatments with higher contents at warm than cold temperature.

Overall, temperature had a significant effect on IAA content in plants grown in compost and on agar with a trend of higher IAA at cold temperature, but this effect was not significant for individual genotypes (Fig. 4g-i). In addition, sucrose increased IAA content on agar medium at warm (*P* = 0.015) and cold temperature (*P* = 0.003) (Additional file 4). GA_3_ content (Fig. 4j-l), on the other hand, was strongly increased by sucrose addition at warm temperature (*P* < 0.001; Additional file 4) but not at cold temperature. The contents of other GAs and of cytokinins are given in Additional file 7.

### Overall changes in hormone, anthocyanin and sugar contents

**Fig. 5:**
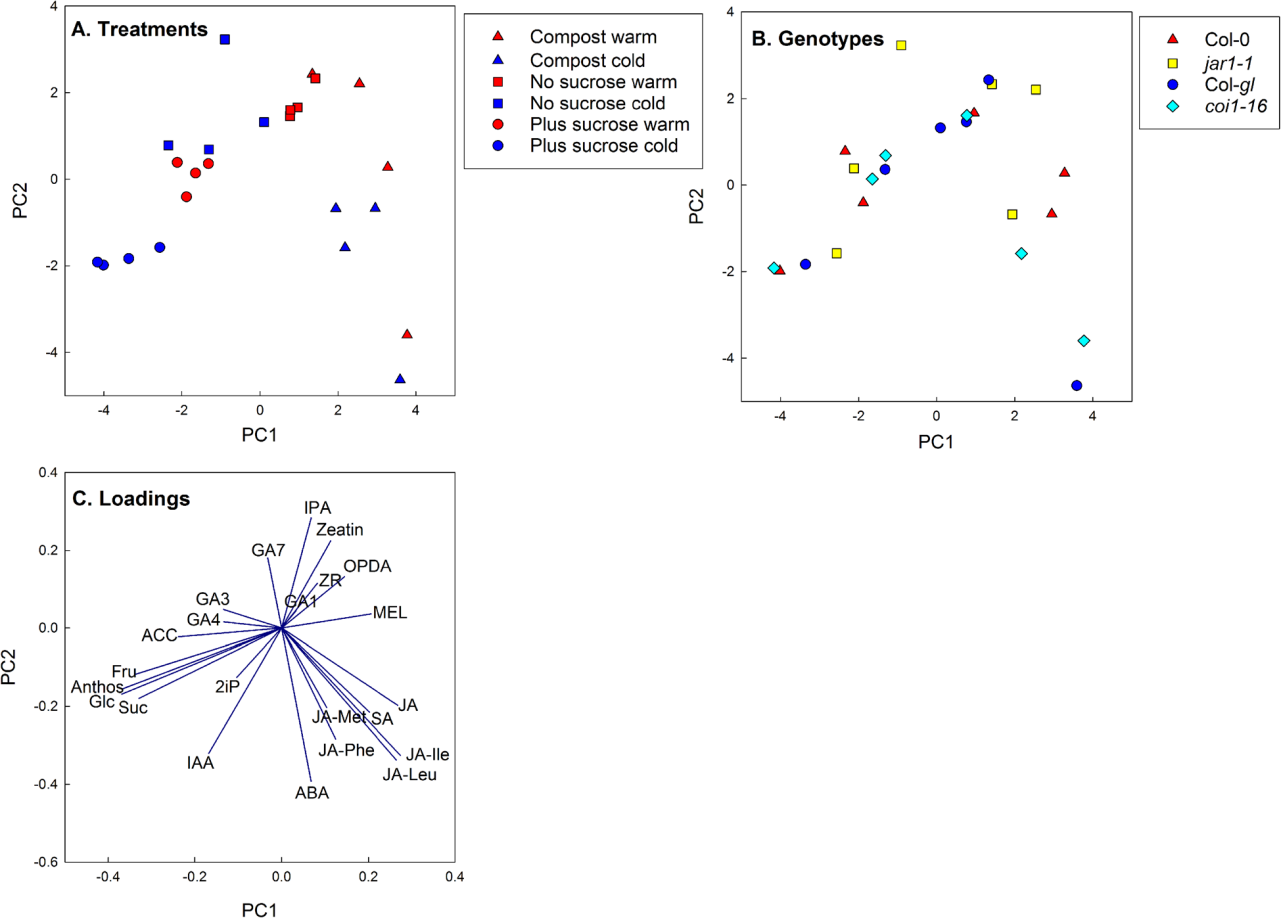
PCA score plots (**a** and **b**) and loading plot (**c**) for the first two principal components. The analysis is based on the contents of all hormones, anthocyanin, glucose, fructose and sucrose (see Additional file 7). The symbols represent treatments (**a**) or genotypes (**b**)

Strong treatment effects were found on anthocyanin, hormone and sugar contents (Figs. 1, 2, 3 and 4). This is reflected in the PCA results (Fig. 5). The score plots of the first two principal components indicate separation by growth medium (compost vs agar), sucrose treatment and temperature (Fig. 5a), but not by genotype (Fig. 5b). PC1 (explaining 26% of the variation) was positively associated with JA, JA-Ile, JA-Leu, SA and melatonin, and negatively with anthocyanin, glucose, fructose and sucrose. PC2 (explaining 17% of the variation) was positively associated with IPA, zeatin and GA_7_, and negatively with ABA and JA-Leu, JA-Ile, JA-Phe and IAA (Fig. 5c). Comparison of the score and loading plots also shows a clear association of cultivation on compost with jasmonates and cultivation on sucrose (especially at low temperature) with sugars and anthocyanins. PC3 (explaining 13% of the variation) mainly separated the cold from the warm treatment (Additional file 8).

### Cold response of the *jar1–1* and *coi1–16* mutants

**Fig. 6:**
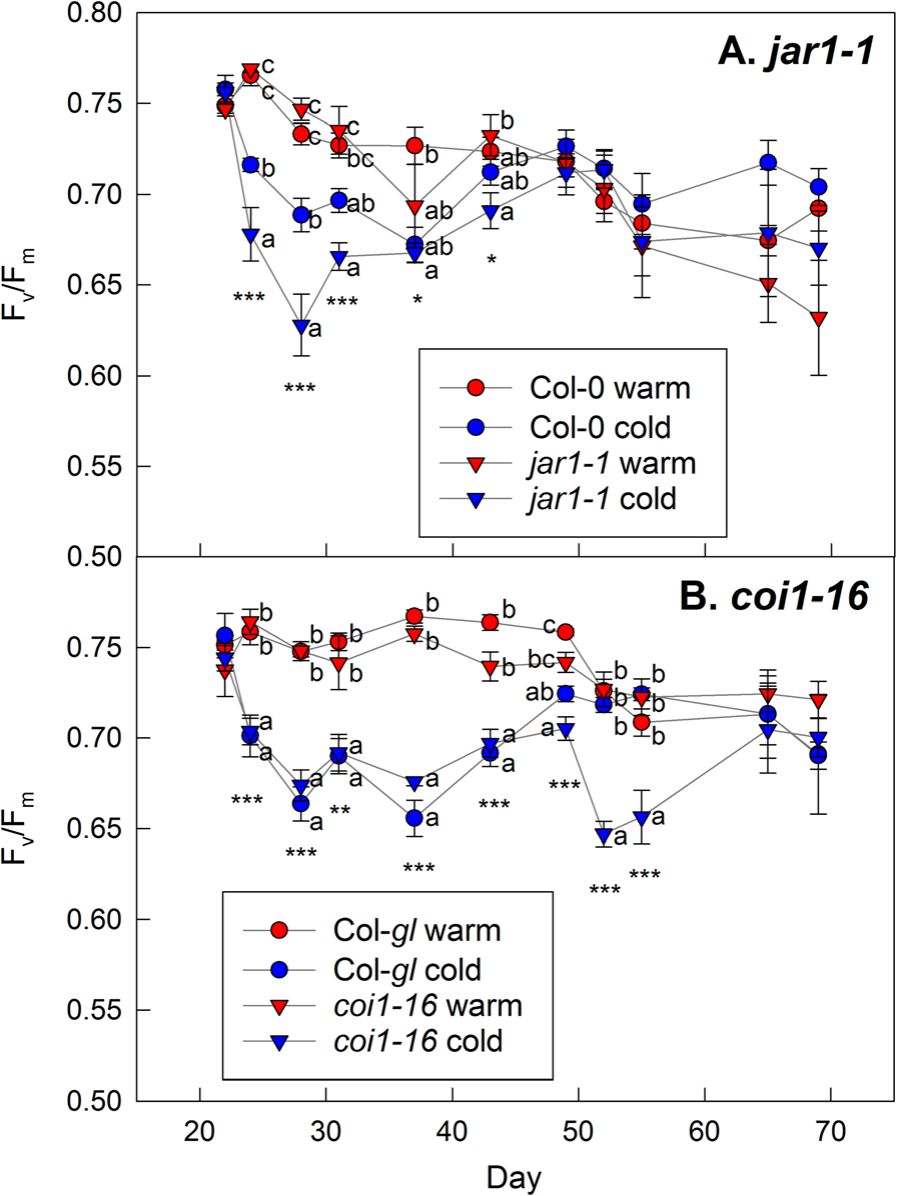
Effect of cold treatment on F_v_/F_m_ in the *jar1–1* (**a**) and *coi1–16* (**b**) mutants and their respective wild types, Col-0 and Col-*gl*. The plants were grown in compost at 20 °C until day 22 and then either kept at 20 °C (red symbols) or transferred to 4 °C (blue symbols) for the remainder of the experiment. F_v_/F_m_ was determined for the whole rosette by fluorescence imaging. Data are means of 5 plants ±SE. The asterisks indicate significant differences for each timepoint (one-way ANOVA; * *P* < 0.05; ** *P* < 0.01; *** *P* < 0.001). Different letters indicate differences between the temperature treatments or genotypes (Tukey’s HSD post-hoc test; *P* < 0.05)

There were no differences in F_v_/F_m_ between wild-type and mutant plants at the end of the experiment (Fig. 1). The ability of the mutants to cold acclimate was confirmed in an independent experiment (Fig. 6). F_v_/F_m_ of the whole rosettes declined in all plants in response to cold treatment. Subsequent recovery of whole-rosette values indicates cold acclimation (Fig. 6), as also demonstrated for agar-grown plants (Additional file 2), although these measurements cannot show if the acclimation happened through formation of cold acclimated new leaves or also acclimation of older leaves. The initial decline was stronger in the *jar1–1* mutant than Col-0 (Fig. 6a), suggesting that it initially suffered more severely from the stress, but the F_v_/F_m_ values then increased to the same value as in wild-types plants, demonstrating that the mutant is not impaired in its ability to cold acclimate. There were no differences in the initial response to cold stress between the *coi1–16* mutant and its Col-*gl* background (Fig. 6b), but the mutant showed a second drop in F_v_/F_m_ later on in development which may have been caused by senescence of the old leaves in combination with delayed formation of new leaves. However, whole-rosette F_v_/F_m_ did subsequently recover. These results suggest subtle effects of low temperature on JA signalling mutants, but also show that they are able to cold acclimate.

## Discussion

Previous work had shown a role for jasmonate signalling in anthocyanin accumulation [19, 28, 29] and for freezing tolerance [10, 11]. It was also shown that jasmonate-induced anthocyanin formation is sucrose dependent [19] and that cold treatment results in sucrose accumulation [2, 8]. Anthocyanin synthesis, in turn, has been related to freezing tolerance [15], suggesting that the jasmonate and sucrose signalling pathways interact. Here, we analysed the interactive effects of sucrose and cold (chilling) stress on anthocyanin formation and hormone contents in wild-type Arabidopsis and in jasmonate signalling mutants.

We found clear impacts of medium type, sucrose availability and temperature on hormone, anthocyanin and sugar contents (Fig. 5). Although the *jar1–1* mutant showed the expected impairment in JA-Ile accumulation (Fig. 3) and compost-grown *coi1–16* plants had increased sugar contents (Fig. 2) and altered hormone responses to temperature (Figs. 3 and 4), disruptions in the cold acclimation pathways were temporary (Fig. 6) and the mutants were able to cold acclimate and accumulate anthocyanins (Fig. 1).

### Hormone contents are affected by the growth medium and temperature

One of the most striking finding was that contents of several stress hormones, JA, JA-Ile, JA-Leu, SA and ABA, were substantially higher in compost than on agar medium (Figs. 3 and 4). Jasmonate synthesis is associated with biotic stress [9], but no signs of pathogen infection or insect infestation were observed, and the plants looked healthy. The formation of defence hormones (jasmonates and SA) could be a response to the microbial environment, which suggests that the contents of these hormones may be considerably higher in nature than under sterile conditions on agar plates. However, other factors (development or nutrient availability) than exposure to microbes may have contributed to differences between agar- and compost-grown plants.

In addition, sucrose addition to the growth medium affected hormone contents, e.g. increasing the contents of JA-Leu, ABA, SA and IAA (Additional file 4). Research on jasmonate signalling has often been carried out using seedlings cultivated with routine addition of sucrose to the growth medium [e.g. 28, 29, 35, 38], which does not allow differentiation between sucrose-dependent and -independent jasmonate responses. At the sucrose concentration of 55 mM (equivalent to 1.88% w/v) that was used in our experiments, sucrose contents exceeded those in plants grown under more natural conditions in compost (Fig. 2). Routine addition of similar concentrations of sucrose to growth medium (e.g. 2% [38] or 3% [29]) may result in unnaturally high sugar contents in the plants that can affect plant responses through interactions between sugar with hormone signalling. In addition, the induction of jasmonates, SA and ABA by sucrose on agar medium may have been related to sucrose-induced senescence, as indicated by a drop in F_v_/F_m_ in the presence of sucrose (Fig. 1c and Additional file 2).

Temperature also has an effect on hormone contents, partially in interaction with sucrose availability. In the alpine perennial, *Arabis alpina*, JA and zeatin contents were increased at low temperature, while IAA content was reduced; however, these effects were genotype-specific [8]. Here, JA-Ile (with and without sucrose addition) and JA-Leu (only with sucrose addition) contents were decreased by the cold treatment (Fig. 3), whereas there was an overall increase in SA and IAA contents in compost in response to cold treatment (Fig. 4). While JA conjugates were not measured in *A. alpina* [8], the effect on IAA content suggests a difference in the response in Arabidopsis compared to the alpine perennial. Differences in changes in hormone contents in response to cold treatment were also found between a winter and spring cultivars of wheat, including a high IAA content in the spring cultivar after long-term cold treatment, which may be linked to lower freezing tolerance [39].

Interactions between temperature and sucrose treatments were observed for GA_3_ which increased in response to sucrose, but only at warm temperature. This is consistent with the role of gibberellins in growth and a reduction in bioactive GAs by the CBF-dependent cold acclimation pathway [40].

### Pathways for the induction of anthocyanin formation by sucrose and cold treatment

Our analysis confirms a close relationship between sugar and anthocyanin contents across the treatments (Fig. 5 and Additional file 6). Since senescence typically results in anthocyanin accumulation, higher anthocyanin content may reflect the early senescence observed after growth on sucrose-containing medium at warm temperature. Sucrose induces anthocyanin accumulation via MYB75 [17, 18], while low temperature probably acts via MYB90 [15]. It was therefore suggested that anthocyanin accumulation at low temperature is not induced by sucrose. Here, cold treatment further increased anthocyanin accumulation in the presence of external sucrose in an additive manner (Fig. 1), despite the observation that there was no cold-related increase in sucrose content when sucrose was supplied externally (Fig. 2). This supports the view that the pathways for sucrose and cold-induced anthocyanin synthesis act independently.

The cold-induced accumulation of anthocyanins in the presence of sucrose could be explained with a decline in gibberellin signalling. Similar to degradation of JAZ proteins in the presence of JA-Ile, GAs induce the degradation of repressors, the DELLA proteins. During cold stress, DELLA proteins accumulate as gibberellins are degraded which results in growth inhibition [40]. A mechanism for crosstalk between the gibberellin and jasmonate signalling pathways that can explain the opposite effects of these groups of hormones in anthocyanin formation has been proposed [33]. By binding to JAZ proteins, DELLA proteins activate jasmonate-responsive genes. Degradation of DELLAs by GAs, on the other hand, represses jasmonate signalling. Moreover, it was recently proposed that GA and JA signalling in anthocyanin synthesis are integrated by the MBW transcriptional complex which includes MYB75 [35]. Sequestration of JAZ proteins by DELLA proteins thus results in MBW-dependent anthocyanin synthesis e.g. during cold stress. This crosstalk of gibberellins and jasmonates is supported by the finding that sucrose-dependent induction of *MYB75* and anthocyanin synthesis were reduced in a quadruple *della* mutant of Arabidopsis [34]. The further induction of anthocyanin formation at cold temperature in the presence of sucrose could therefore be a result of lower contents of active GAs, such as GA_3_ at cold compared to warm temperature (Fig. 4). Such a mechanism could also explain how the DELLA pathway could activate jasmonate signalling downstream of the COI1/JA-Ile pathway in the mutants (see below).

### The *jar1–1* and *coi1–16* mutants have altered hormone contents

Accumulation of ABA in response to water deficit was much lower in the *jar1–1* and *coi1–16* mutants than wild-type plants [31], suggesting that jasmonate signalling acts upstream of ABA. In contrast, it was proposed for tomato that JA acts downstream of ABA in cold signalling [41]. ABA contents were not reduced in the mutants in response to cold stress, although the *coi1–16* mutant showed unusual patterns of ABA accumulation after growth on compost (Fig. 4). In addition to ABA, the *coi1–16* mutant contained increased amounts of JA-Leu and JA-Ile compared to its wild type after growth in compost at warm temperature. SA and IAA contents, on the other hand, were reduced compared to wild type at low temperature. This suggests interactions of altered jasmonate signalling with hormone synthesis pathways. Moreover, sugar contents were significantly increased in the *coi1–16* mutant in compost (Fig. 2).

The *jar1–1* mutant was unable to accumulate JA-Ile and JA-Leu under any of the conditions tested, but did contain small amounts of JA-Ile (Fig. 3). This is in agreement with previous reports of some JA-Ile being formed in the *jar1–1* mutant [21, 22] and indicates that there may be another enzyme that can catalyse the formation of JA conjugates. The K_m_ value of the JAR1 enzyme for Ile is considerably lower than its K_m_ for Leu, Val and Phe, suggesting that it primarily catalyses the conjugation of JA with Ile [22]. Our results do, however, suggest that JAR1 is also involved in JA-Leu synthesis. Accumulation of the precursor of jasmonates, OPDA, on agar medium at cold temperature (Fig. 3) indicates a feedback effect of lower contents of the JA conjugates in the *jar1–1* mutant.

### No evidence for an essential role of jasmonate signalling in anthocyanin accumulation and cold acclimation

In addition to impaired ABA formation during drought stress [31], both the *jar1–1* and *coi1–16* mutants have been shown to be hypersensitive to ozone [32, 42], supporting the view that these mutant alleles are impaired in stress responses. Low temperature treatment results in JA accumulation, and application of external JA can increase freezing tolerance. In addition, *jar* and *coi1* mutants showed decreased freezing tolerance with and without cold acclimation [10]. However, their physiological response to low, but above-freezing (chilling) temperatures was not explored. While we detected differences in the development of cold acclimation (Fig. 6), both mutants were able to acclimate and reached normal F_v_/F_m_ values. In addition, anthocyanin accumulation in response to cold (Fig. 1) or sucrose treatment (Additional file 3) was not impaired. However, our results do not allow us to draw direct conclusions concerning freezing tolerance.

The ability of the *jar1–1* mutant to accumulate anthocyanins is in agreement with previous findings for jasmonate and sucrose responses in this mutant [19, 30]. Normal sucrose-dependent induction of anthocyanin formation in *jar1–1* could be explained with the presence of small amounts of JA-Ile (Fig. 3; see also [21, 22]). However, the inability of the *jar1–1* mutant to accumulate JA-Ile in response to sucrose makes it unlikely that JA-Ile synthesis is required for increased anthocyanin content. In our experiments, JA-Ile and anthocyanin contents were negatively (and not positively) correlated (Additional file 6), supporting the view that JA-Ile accumulation is not required for the induction of anthocyanin formation in response to sucrose and low temperature.

Normal anthocyanin contents in the *coi1–16* mutant contradict the previous view that, in contrast to the upstream signalling component JAR1, COI1 is required for sucrose-dependent anthocyanin formation [19]. The observation that anthocyanins accumulated in the *coi1–16* mutant here can possibly be explained with the fact that *coi1–16*, in contrast to *coi1–1*, is leaky. While *coi1–1* is male sterile, *coi1–16* produces fertile pollen at a temperature of 16 °C (but not at 22 °C) and can therefore be maintained as a homozygous line [30]. To overcome the problem of male sterility, the previous study [19] selected homozygous *coi1–1* seedlings on JA-containing medium and performed experiments on leaf strips, showing that expression of the dihydroflavonol reductase (*DFR*) gene for anthocyanin synthesis was not induced by sucrose, MeJA, or a combination of both, in *coi1–1*. However, anthocyanin content itself was not measured in *coi1–1* and gene expression changes were reported after 24 h, whereas treatments were much longer here. It has been described that the *coi1–1* mutant allele accumulates anthocyanins normally when plants are germinated under continuous light or exposed to water deficit [23], supporting our findings that jasmonate signalling does not play a major role in anthocyanin accumulation, as shown here for cold stress, despite effects on the regulation of genes involved in anthocyanin synthesis as demonstrated earlier [19].

Other experiments showing a role of jasmonate signalling in anthocyanin formation, e.g. using the leaky *coi1–2* mutant allele, were mainly conducted in seedlings [28, 29]. Since the anthocyanin contents presented here are the endpoint of cold-treatment of mature plants for 15 (in compost) or 25 (on agar plates) days, even a lower initial rate of anthocyanin accumulation may have resulted in normal final contents in the *coi1–16* mutant. In the presence of sucrose, when GA_3_ content was reduced by cold temperature (Fig. 4), antagonistic effects between gibberellin and jasmonate signalling through sequestration of JAZ proteins by DELLA [33] could activate jasmonate signalling. As this interaction occurs downstream of the JA-Ile/COI1 pathway, it could compensate for the *jar1–1* and *coi1–16* mutations under cold conditions, and thus result in anthocyanin accumulation in response to sucrose.

If JAR1 is required upstream of COI1, then *jar1* mutants should show the same defects and increased stress responses as *coi1* mutants. However, the *jar1–1* mutant accumulated the precursor of JAs, OPDA, on agar medium at cold temperature (Fig. 3). OPDA is proposed to act independently of jasmonate signalling [26] which could potentially explain normal anthocyanin formation in this mutant. However, a new pathway of JA synthesis from OPDA was recently discovered, which supports the view that OPDA effects are mediated by JA formation and signalling through the JA-Ile/COI1 pathway [43]. Overall, it is unlikely that sugar-induced anthocyanin synthesis would require COI1, but not JA-Ile formation by JAR1.

## Conclusions

Reduced signalling via the JA-Ile/COI1 pathway does not impair long-term anthocyanin accumulation in response to cold stress or sucrose treatment. Since the *jar1–1* mutant does contain low amounts of JA-Ile and *coi1–16* mutant is leaky, it cannot be excluded that low levels of JA-Ile or COI1 are required for anthocyanin accumulation, but a drop in GA_3_ in response to low temperature in the presence of sucrose may have compensated for impaired jasmonate signalling. In addition, reduced jasmonate signalling does not severely impair the ability of plants to cold acclimate. Large effects on hormone contents were found not only in response to cold treatment, but also dependent on sucrose availability and growth on agar compared to growth in compost. Overall, the results presented here demonstrate that research on hormone signalling pathways in stress tolerance needs to take other external factors, such as media composition, into account and that adjustments in the contents of other hormones in the mutants may underlie flexibility of cold stress responses.

## Methods

### Plant material and growth conditions

The *jar-1-1* mutant was obtained from the Nottingham Arabidopsis Stock Centre. Mutation of the *JAR1* gene was confirmed by sequencing. The *coi1–16* mutant and Col-*gl* background line were provided by Alessandra Devoto (Royal Holloway, University of London). Since the original *coi1–16* mutant harboured a second mutation [44], the mutant line used here had been cleaned up of additional mutations by back-crossing. Both the *jar1–1* and *coi-16* mutants showed normal development on medium containing 25 μM MeJA, whereas wild-type growth was stunted with high accumulation of anthocyanins (Additional file 9).

Seeds were placed onto moist John Innes No.2 compost (Levington) and stratified for 3 days at 5 °C before cultivation at 20 °C (warm treatment) and a photon flux density of 120 μmol m^− 2^ s^− 1^ in a 12 h day/12 h night cycle. After 39 days, half of the plants were transferred into 4 °C (cold treatment) under the same light conditions to impose cold stress. After 15 days of temperature treatment, leaves were harvested at mid-day into liquid nitrogen for sugar, anthocyanin and hormone analysis. Five plants in separate pots were grown per genotype and treatment. An independent experiment was conducted to confirm the impact of cold stress on anthocyanin content in the mutants (Additional file 5).

For cultivation on agar, the seeds (about 10 seeds per plate) were sown onto 1% agar in quarter-strength NH_4_NO_3_-free MS medium (Duchefa Biochemie) containing 4.7 mM nitrate, with or without addition of 55.5 mM sucrose. After stratification, the agar plates were transferred into 20 °C (warm treatment) at a photon flux density of 120 μmol m^− 2^ s^− 1^ in a 12 h day/12 h night cycle. After 13 days of growth under these conditions, half of the plates were transferred into 4 °C (cold treatment), with five replicate plates per line and treatment. The plates were arranged in a randomised block design. The plants were subjected to the temperature treatments for 21 days before harvest of the shoots at mid-day for sugar analysis. In addition, shoots were harvested after 25 days of temperature treatment for anthocyanin and hormone analysis.

### Determination of maximum photosystem II efficiency

Maximum photosystem II efficiency (F_v_/F_m_) was determined in individual leaves (leaf 6) of plants grown in compost and in whole leaf rosettes of compost- and agar-grown plants as in [36]. For individual leaf measurements, an FMS2 chlorophyll fluorometer (Hansatech) was used; whole-rosette measurements were performed with a FluorCam imaging fluorometer (Photon Systems Instruments). Plants on agar plates were analysed individually and plate averages were calculated. To avoid pseudoreplication, the averages for the separate plates were then used as replicates in the statistical analysis (five plates per genotype and treatment). If not otherwise indicated, F_v_/F_m_ was determined a day before the harvest for anthocyanin analysis.

### Analysis of sugar, anthocyanin and hormone contents

Sugars were extracted in 80% ethanol at 80 °C and determined spectrophotometrically using a coupled enzymatic assay [45]. Total anthocyanins were determined as described by [46]. In short, 50 mg plant material was extracted in 1.5 mL methanol using ultrasonication and vortexing. The extracts were centrifuged at 13000 rpm for 10 min at 4 °C. The pellet was re-extracted following the same procedure. Supernatants were collected and pooled in order to acidify the extracts by adding 1% HCl. Then total anthocyanins were measured spectrophotometrically at 530 nm using the molar extinction coefficient of cyanidin-3- glucoside as a reference.

The endogenous plant hormones were extracted and quantified by UHPLC/ESI-MS/MS as described [47, 48], including jasmonic acid (JA), its precursor 12-*oxo*-phytodienoic acid (OPDA) and its amino acid conjugates JA-isoleucine (JA-Ile), JA-leucine (JA-Leu), JA-phenylalanine (JA-Phe), JA-valine (JA-Val) and JA-methionine (JA-Met), abscisic acid (ABA), salicylic acid (SA), the auxin indole-3-acetic acid (IAA), melatonin, the cytokinins *tran*s-zeatin (*t*-Z), its riboside (*t*-ZR), isopentenyl adenosine (IPA) and 2-isopentenyl adenine (2iP), and the gibberellins GA_1_, GA_3_, GA_4_ and GA_7_. Deuterium-labelled compounds were used as internal standards.

### Statistical analysis

Five replicates (individual plants in compost or replicate agar plates) were analysed for each treatment and time point. Data were analysed by one-way or two-way analysis of variance (ANOVA) as indicated in the figure legends using the SPSS 20.0 statistical package. Multiple comparison tests were carried out with Tukey’s HSD post-hoc test. Correlation and principal component analysis (PCA) were performed with Minitab 16. In all cases, differences were considered significant at a probability level of *P* < 0.05.

## Supplementary information

**Additional file 1 **Effect of cold treatment on F_v_/F_m_ in individual leaves (leaf position 6) of the *jar1–1* and *coi1–16* mutants and their respective wild types, Col-0 and Col-*gl* grown in compost.

**Additional file 2 **Effect of cold treatment on F_v_/F_m_ in the *jar1–1* and *coi1–16* mutants and their respective wild types, Col-0 and Col-*gl* grown on agar.

**Additional file 3 **Effect of sucrose treatment on F_v_/F_m_ and anthocyanin content in the *jar1–1* and *coi1–16* mutants and their respective wild types, Col-0 and Col-*gl*.

**Additional file 4.** Two-way ANOVA testing for sucrose effects, genotype effects and interactions between sucrose treatment and genotype.

**Additional file 5 **Effect of cold treatment on anthocyanin content in the *jar1–1* and *coi1–16* mutants and their respective wild types, Col-0 and Col-*gl*.

**Additional file 6.** Correlation between hormones, anthocyanins and sugars.

**Additional file 7 **Hormone, anthocyanin and sugar contents in Col-0, *jar1–1*, Col-*gl* and *coi1–16* plants grown at cold (4 °C) or warm (20 °C) temperature.

**Additional file 8.** PCA score plots and loading plot for the first and third principal components.

**Additional file 9 **Response of the *jar1–1* and *coi1–16* mutants to jasmonate treatment.

## Data Availability

The datasets supporting the conclusions of this article are included in Additional file 7 and any other datasets used in this study are available from the corresponding author on reasonable request.

## Abbreviations

ABA: Abscisic acid
*coi*: coronatine insensitive
GA: Gibberellin
IAA: Indole-3-acetic acid
JA: Jasmonic acid
*jar*: jasmonate resistant
OPDA: 12-*oxo*-phytodienoic acid (OPDA)
SA: Salicylic acid
