# Supplementary material for: Interactions between sucrose and jasmonate signalling in the response to cold stress
Source: BMC Plant Biol. 2020 Apr 22;20:176. doi: 10.1186/s12870-020-02376-6 (PMC7178619; doi:10.1186/s12870-020-02376-6)
Supplement: Supplementary file 1 — Additional file 1 Effect of cold treatment on Fv/Fm in individual leaves (leaf position 6) of the jar1–1 and coi1–16 mutants and their respective wild types, Col-0 and Col-gl grown in compost. [file 12870_2020_2376_MOESM1_ESM.pdf]

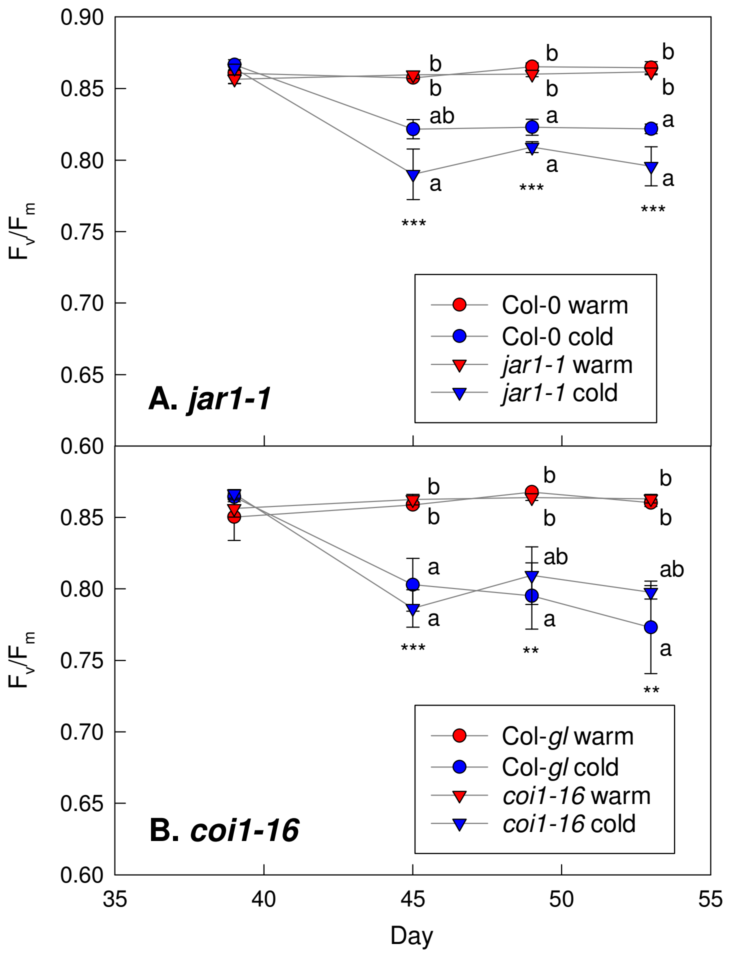

Additional file 1. Effect of cold treatment on F_v/F_m in individual leaves (leaf position 6) of the *jar1-1* (A) and *coi1-16* (B) mutants and their respective wild types, Col-0 and Col-*gl*. The plants were grown in compost at 20°C until day 39 and then either kept at 20°C (red symbols) or transferred to 4°C (blue symbols) for the remainder of the experiment. Data are means of 5 plants ±SE. The asterisks indicate significant differences for each timepoint (one-way ANOVA; * $P<0.05$; ** $P<0.01$; *** $P<0.001$). Different letters indicate differences between the temperature treatments or genotypes (Tukey's HSD post-hoc test; $P<0.05$).
