# Supplementary material for: Interactions between sucrose and jasmonate signalling in the response to cold stress
Source: BMC Plant Biol. 2020 Apr 22;20:176. doi: 10.1186/s12870-020-02376-6 (PMC7178619; doi:10.1186/s12870-020-02376-6)
Supplement: Supplementary file 2 — Additional file 2 Effect of cold treatment on Fv/Fm in the jar1–1 and coi1–16 mutants and their respective wild types, Col-0 and Col-gl grown on agar. [file 12870_2020_2376_MOESM2_ESM.pdf]

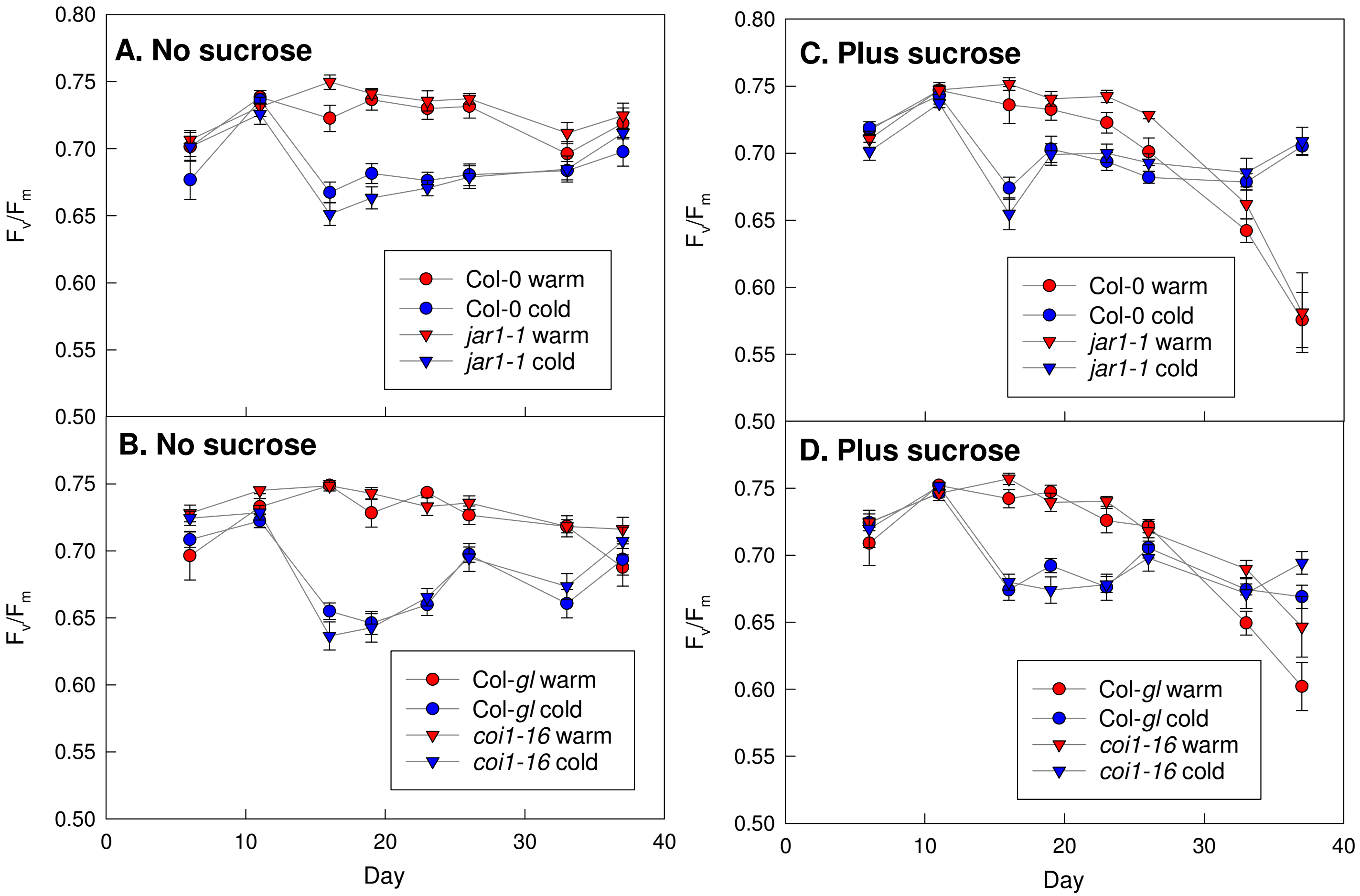

Additional file 2. Effect of cold treatment on F_v/F_m in the *jar1-1* (A and C) and *coi1-16* (B and D) mutants and their respective wild types, Col-0 and Col-*gl*. The plants were grown on agar plates without sucrose (A and B) or with addition of 55 mM sucrose (C and D). F_v/F_m was determined for the whole rosette by fluorescence imaging. The plates were incubated at 20°C until day 13 and then either kept at 20°C (red symbols) or transferred to 4°C (blue symbols) for the remainder of the experiment. Data are means of 5 plates ±SE.
