# Supplementary material for: Interactions between sucrose and jasmonate signalling in the response to cold stress
Source: BMC Plant Biol. 2020 Apr 22;20:176. doi: 10.1186/s12870-020-02376-6 (PMC7178619; doi:10.1186/s12870-020-02376-6)
Supplement: Supplementary file 3 — Additional file 3 Effect of sucrose treatment on Fv/Fm and anthocyanin content in the jar1–1 and coi1–16 mutants and their respective wild types, Col-0 and Col-gl. [file 12870_2020_2376_MOESM3_ESM.pdf]

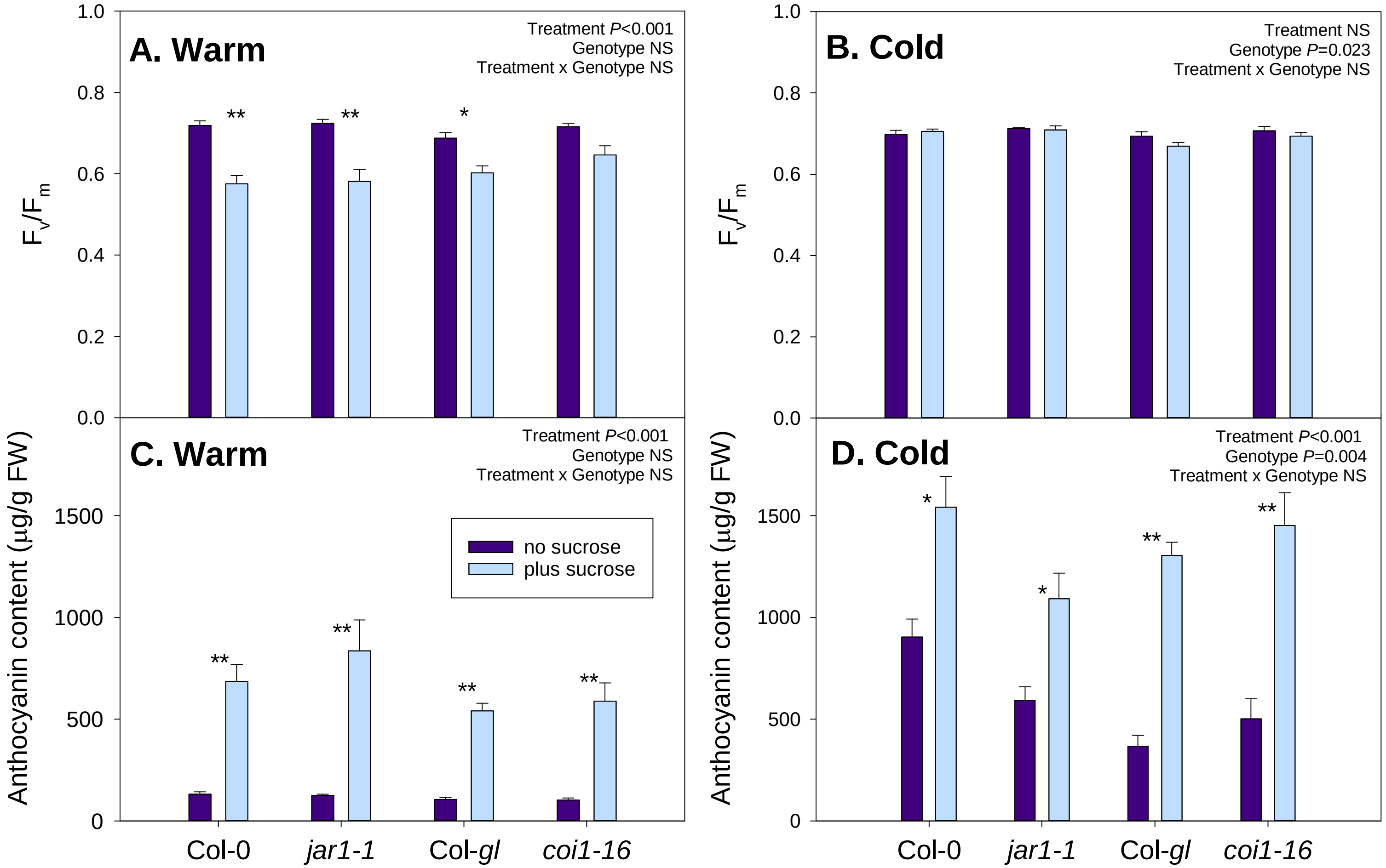

Additional file 3. Effect of sucrose treatment on F_v/F_m (A and B) and anthocyanin content (C and D) in the *jar1-1* and *coi1-16* mutants and their respective wild types, Col-0 and Col-*gl*. The plants were grown on agar plates without sucrose (dark blue bars) or with addition of 55 mM sucrose (light blue bars) at warm (20°C; A and C) or cold temperature (4°C; B and D). The plants were harvested after 25 days of treatment (age of plants 38 days) for anthocyanin analysis. F_v/F_m was measured on the day before the harvest. Data are means of plants from 5 plates +SE. Sucrose treatment effects, genotype effects and interactions were analysed by two-way ANOVA. The asterisks indicate statistically significant differences between the no sucrose and plus sucrose treatments for each genotype (Tukey's HSD post-hoc test;* $P<0.05$; ** $P<0.01$).
