# Supplementary material for: Interactions between sucrose and jasmonate signalling in the response to cold stress
Source: BMC Plant Biol. 2020 Apr 22;20:176. doi: 10.1186/s12870-020-02376-6 (PMC7178619; doi:10.1186/s12870-020-02376-6)
Supplement: Supplementary file 5 — Additional file 5 Effect of cold treatment on anthocyanin content in the jar1–1 and coi1–16 mutants and their respective wild types, Col-0 and Col-gl. [file 12870_2020_2376_MOESM5_ESM.pdf]

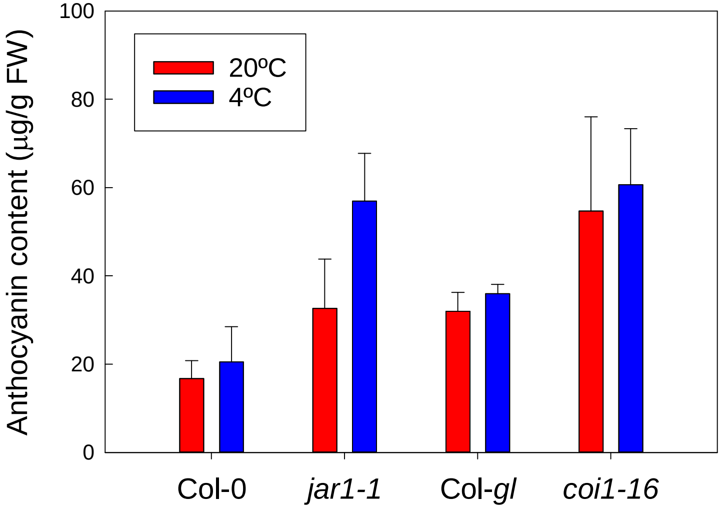

Additional file 5. Effect of cold treatment on anthocyanin content in the *jar1-1* and *coi1-16* mutants and their respective wild types, Col-0 and Col-*gl*. The plants were grown in compost at warm temperature for 39 days before temperature treatment of 27 days at warm temperature (red bars) or cold temperature (blue bars). Data are means of 5 plants +SE.
