# Supplementary material for: Interactions between sucrose and jasmonate signalling in the response to cold stress
Source: BMC Plant Biol. 2020 Apr 22;20:176. doi: 10.1186/s12870-020-02376-6 (PMC7178619; doi:10.1186/s12870-020-02376-6)
Supplement: Supplementary file 8 — Additional file 8. PCA score plots and loading plot for the first and third principal components. [file 12870_2020_2376_MOESM8_ESM.pdf]

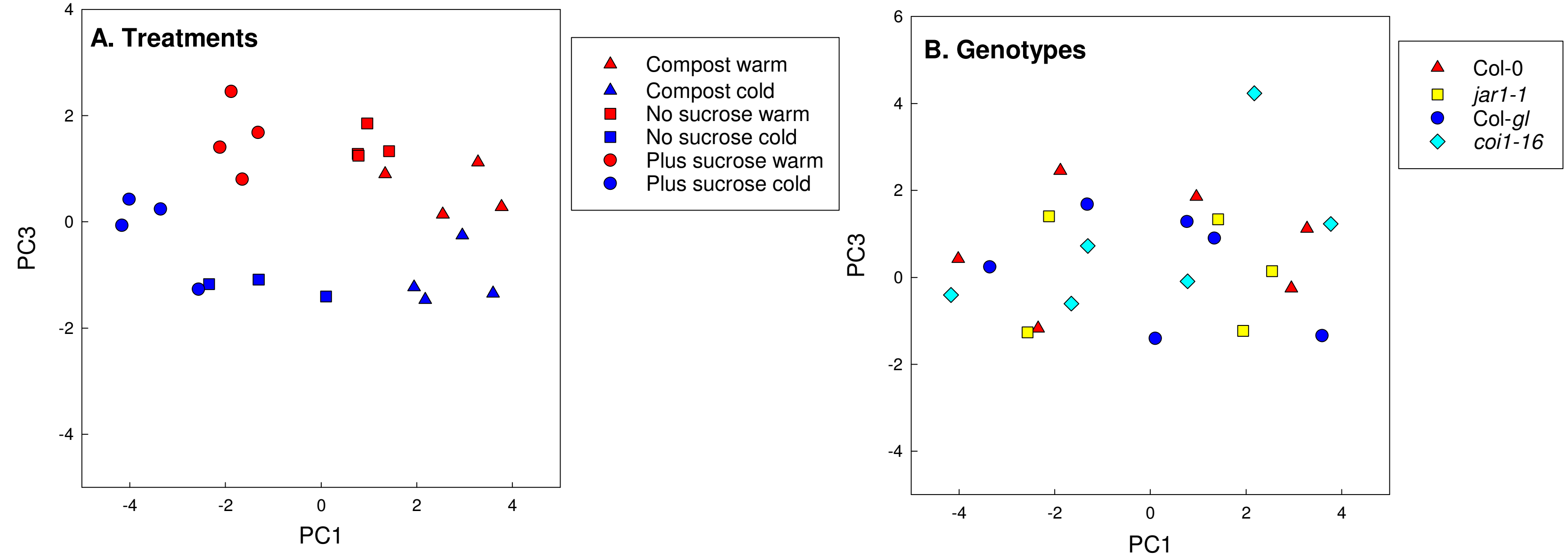

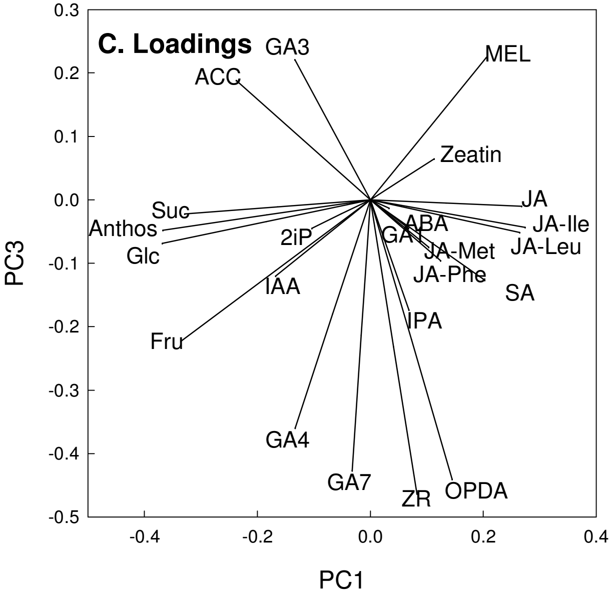

Additional file 8. PCA score plots (A and B) and loading plot (C) for the first and third principal components. The analysis is based on the contents of all hormones, anthocyanin, glucose, fructose and sucrose (see Supplemental Table S1). The symbols represent treatments (A) or genotypes (B).
