# Supplementary material for: Interactions between sucrose and jasmonate signalling in the response to cold stress
Source: BMC Plant Biol. 2020 Apr 22;20:176. doi: 10.1186/s12870-020-02376-6 (PMC7178619; doi:10.1186/s12870-020-02376-6)
Supplement: Supplementary file 9 — Additional file 9 Response of the jar1–1 and coi1–16 mutants to jasmonate treatment. [file 12870_2020_2376_MOESM9_ESM.pdf]

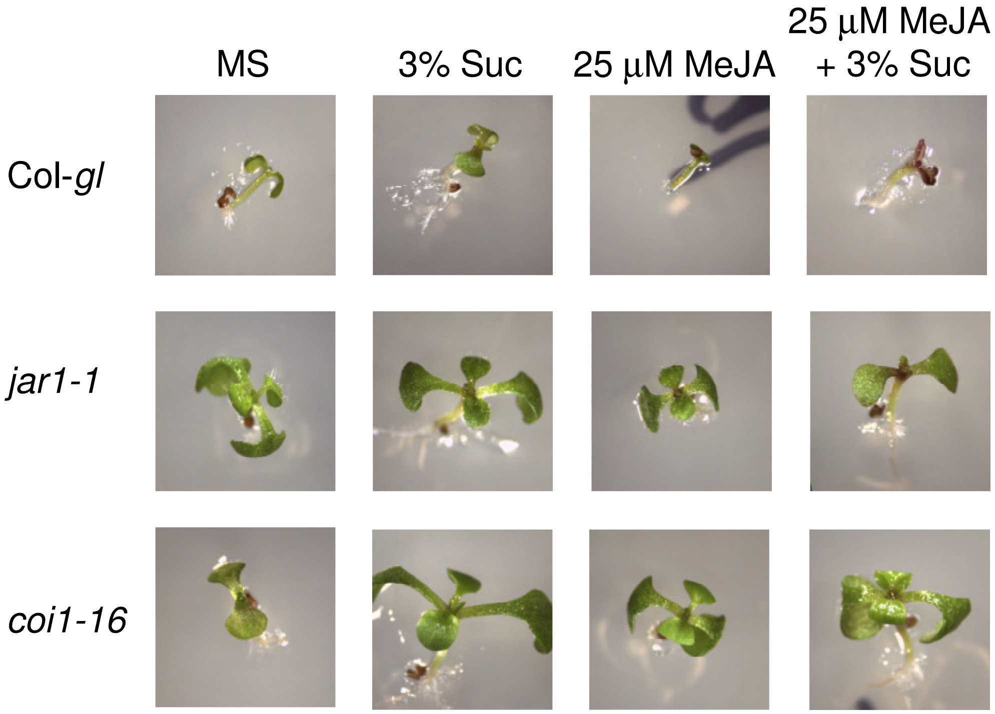

Additional file 9. Response of the the *jar1-1* and *coi1-16* mutants to jasmonate treatment. The plants were grown on MS medium with or without addition of 3% sucrose and/or 25 μM methyl jasmonate.
